# Changes in voxel-wise gray matter asymmetry over time

**DOI:** 10.3389/fnins.2025.1671341

**Published:** 2025-12-01

**Authors:** Florian Kurth, Nicolas Cherbuin, Christian Gaser, Eileen Luders

**Affiliations:** 1 School of Psychology, University of Auckland, Auckland, New Zealand; 2 Department of Diagnostic and Interventional Radiology, Jena University Hospital, Jena, Germany; 3 National Centre for Epidemiology and Population Health, Australian National University, Canberra, ACT, Australia; 4 Department of Neurology, Jena University Hospital, Jena, Germany; 5 Department of Psychiatry and Psychotherapy, Jena University Hospital, Jena, Germany; 6 German Center for Mental Health (DZPG), partner site Jena-Magdeburg-Halle, Halle, Germany; 7 Department of Women’s and Children’s Health, Uppsala University, Uppsala, Sweden; 8 Laboratory of Neuro Imaging, School of Medicine, University of Southern California, Los Angeles, CA, United States

**Keywords:** age, asymmetry, brain, gray matter, sex

## Abstract

Hemispheric brain asymmetries emerge in early life but continue to change over time. However, there is no consensus on whether asymmetries become weaker or stronger with age or which brain regions are most affected. Here, we set out to further explore age-related changes in brain asymmetry, with a particular focus on voxel-wise gray matter asymmetry. For this purpose, we selected a sample of 2,322 participants (1,150 women/1,172 men), aged between 47 and 80 years (mean 62.3 years), from the UK Biobank. Each participant was scanned twice; with an interval between baseline and follow-up scans ranging between 1 and 7 years (mean 2.4 years). Significant changes in asymmetry were observed, particularly in the temporal and occipital lobe, as well as the cerebellum. Overall, decreases in asymmetry were more prominent than increases, but with hemisphere-specific effects (i.e., leftward asymmetries decreased more than increased, while rightward asymmetries increased more than decreased). Changes in asymmetry were not significantly associated with chronological age or biological sex, suggesting that these changes neither accelerate nor decelerate with increasing age, and do not differ between the sexes. Follow-up research – potentially incorporating additional morphometric measures, different stages of life, and/or clinical populations – is necessary, not only to replicate the current findings but also to investigate changes over longer timeframes.

## 1 Introduction

Structural and functional hemispheric differences are evident early in life and have been shown to change across the lifespan (Kong et al., 2022; Ocklenburg and Gunturkun, 2018; Ocklenburg and Gunturkun, 2024a; Ocklenburg et al., 2024; Toga et al., 2009). Although various theories have been proposed to explain age-related changes in asymmetry, the precise nature of these changes remains poorly understood. One perspective suggests a gradual recruitment of homotopic contralateral regions with age, potentially leading to decreased asymmetry (Cabeza, 2002). Alternatively, asymmetry may increase due to asymmetric atrophy, with one hemisphere undergoing more rapid degeneration than the other (Thompson et al., 2003).

The outcomes of existing studies are mixed; some report significant changes in asymmetry with increasing age, while others find no such effect (Guadalupe et al., 2017; Kong and Francks, 2022; Kong et al., 2018; Minkova et al., 2017; Ocklenburg and Gunturkun, 2024a; Smeets et al., 2010; Thompson et al., 2003). When asymmetry change is reported, there is little agreement on its nature, such as whether asymmetry increases, decreases, or even reverses direction (from rightward to leftward or from leftward to rightward, respectively). Moreover, it is not clear whether asymmetry change is driven by gain in the right hemisphere, loss in the left hemisphere (or vice versa), or a combination of both. Furthermore, while there is general agreement that asymmetry does not change uniformly across the brain, reports differ with respect to the specific region undergoing change.

The present study was designed to characterize changes in voxel-wise gray matter asymmetry using longitudinal data from more than 2,000 participants. Specifically, we investigated whether brain asymmetry changes over time and whether any changes in brain asymmetry are impacted by chronological age and by biological sex. Altogether, this approach allowed us to disentangle complex patterns of asymmetry change and assess whether age and sex moderate these trajectories in a regionally specific manner.

## 2 Methods

### 2.1 Data and sample

All data were obtained from the UK Biobank (https://www.ukbiobank.ac.uk; application #47813). The UK Biobank holds the ethical approval from the North West Multi-Centre Research Ethics Committee (MREC) and is in possession of the informed consents. Additional approval was provided by the University of Auckland, New Zealand (protocol #020825). The current study was based on T1-weighted brain images, which were acquired at three different sites on a 3T Siemens Skyra scanner using a 32-channel head coil, as described elsewhere (http://biobank.ctsu.ox.ac.uk/crystal/crystal/docs/bmri_V4_23092014.pdf; Alfaro-Almagro et al., 2018). Individual datasets that did not include a follow-up brain scan or information on age, sex, site, and/or handedness were removed. Moreover, all participants with neuropsychiatric or neurological conditions, as well as history of stroke or cancer were excluded. The final sample included 2,322 participants (1,150 women/1,172 men), ranging in age between 47 and 80 years (mean ± SD: 62.25 ± 7.35 years). Each participant was scanned twice, with the interval between baseline and follow-up scans ranging between 1 and 7 years (mean ± SD: 2.39 ± 0.82 years). Out of that final sample 2,100 participants (90%) were right-handers, reflecting the expected distribution of handedness in the population (Annett, 1973).

### 2.2 Data processing

The analyses were conducted using voxel-based morphometry (Ashburner and Friston, 2000; Kurth et al., 2015b) and running the longitudinal workflow for age effects (Gaser et al., 2024), as implemented in CAT12 (version 12.8; https://neuro-jena.github.io/cat/) and SPM12 (version r7771; https://www.fil.ion.ucl.ac.uk/spm/). The T1-weighted images were processed, as detailed elsewhere (Gaser et al., 2024). Briefly, all images acquired at baseline and follow-up were spatially aligned between these two time points using rigid body transformations, corrected for magnetic field inhomogeneities, classified as gray matter (GM), white matter (WM), and cerebrospinal fluid (CSF), and spatially normalized at a resolution of 1.5 × 1.5 × 1.5 mm^3^ using linear transformations and non-linear warping. The normalized GM segments were then modulated by the Jacobian determinant derived from the normalization matrix to preserve the original voxel-wise GM (Ashburner and Friston, 2000; Kurth et al., 2015b). The resulting modulated normalized GM segments were then flipped along the *x*-axis, and both original and flipped tissue segments were warped to a symmetric Shooting Template in MNI space and modulated again (Kurth et al., 2015a). Altogether, this ensured a voxel-wise comparability of local gray matter between hemispheres, across participants, as well as between time points.

### 2.3 Calculation of the asymmetry index

For both baseline and follow-up segments, the asymmetry index (AI) was calculated at each voxel as AI = (right − left) / [0.5 × (right + left)]. This was followed by discarding duplicate information in the left hemisphere. The voxel-wise AI values within the remaining right hemisphere were smoothed using an 8 mm FWHM kernel (Kurth et al., 2015a). The smoothed voxel-wise AI values at baseline (AI_BASELINE_) served as dependent variable for statistical analysis I (see Section 2.4). In addition, voxel-wise change maps were calculated by subtracting the smoothed voxel-wise AI values at baseline from the smoothed voxel-wise AI values at follow-up (AI_FOLLOW-UP_ – AI_BASELINE_). These smoothed voxel-wise change values served as dependent variable for statistical analysis II (see Section 2.4).

### 2.4 Statistical analysis

All statistical analyses were performed in Matlab and SPM12 (version r7771) using general linear models. Analysis I served to map asymmetry at baseline, with the voxel-wise AI_BASELINE_ values as dependent variable and the intercept as the variable of interest. Age, age^2^, sex, total intracranial volume – TIV (determined as the sum of GM, WM, and CSF), and scanner site were treated as variables of no interest. Analysis II served to map changes in asymmetry over time and additionally tested whether the rate or pattern of these changes varied with age or between men and women. Dependent variables here were the voxel-wise AI_FOLLOW-UP_ – AI_BASELINE_ values, whereas independent variables were the intercept, age, and sex. Variables of no interest were TIV, scanner site, as well as the duration between the two time points. All findings pertaining to analysis I and analysis II were corrected for multiple comparisons on cluster level by controlling the family-wise error at *p* ≤ 0.05 (Friston et al., 1996; Kurth et al., 2015a), which was achieved by using a cluster-forming threshold at *p* ≤ 0.001 and correcting for non-stationarity (Hayasaka et al., 2004).

### 2.5 Visualization

The significance clusters were superimposed on a template in the symmetric MNI space (the same template used for symmetric spatial normalization; see Section 2.4). To visualize significant asymmetry at baseline (analysis I), significant rightward asymmetry (positive AI) was projected onto the right hemisphere, whereas significant leftward asymmetry (negative AI) was projected onto the left hemisphere (the latter was achieved by flipping these clusters onto the left hemisphere). To visualize the significant change in asymmetry over time (analysis II), the cluster-specific mean AI was extracted at baseline (to determine the initial asymmetry) and at follow-up (to determine a possible switch in asymmetry over time), as described elsewhere (Kurth et al., 2015a; Kurth et al., 2024). Significant changes of rightward asymmetry (positive AI at baseline) were projected onto the right hemisphere, and significant changes in leftward asymmetry (negative AI at baseline) onto the left hemisphere (the latter, again, by flipping clusters onto the left hemisphere).^1^ In addition, for analysis II, all significant clusters were transformed to the *MNI152NLin2009cAsym* space to be able to report the cluster-specific MNI coordinates.

## 3 Results

### 3.1 Asymmetry at baseline (analysis I)

**Figure 1:**
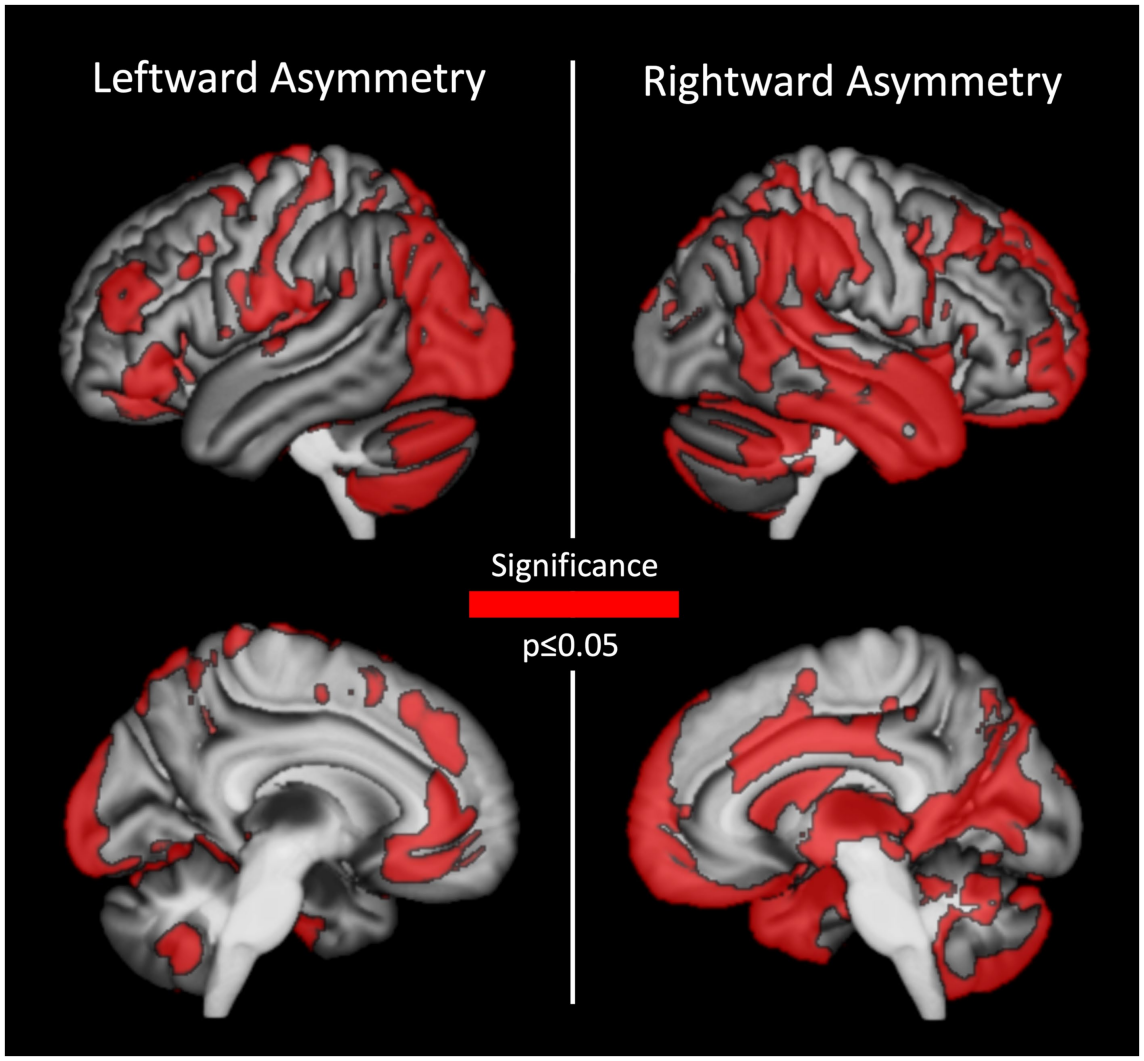
Regions with significant gray matter asymmetry at baseline. Leftward asymmetries are shown on the left, rightward asymmetries are depicted on the right.

As shown in Figure 1, almost all regions of the brain show either a significant rightward- or leftward asymmetry (i.e., hardly any region is symmetric). More specifically, there were two clusters with a significant rightward asymmetry covering large parts of the hemisphere: the first cluster comprised 63,223 voxels (*p* ≤ 0.001, FWE-corrected on cluster-level) covering most of the hemisphere, while the second cluster comprised 2,217 voxels (*p* = 0.004, FWE-corrected on cluster-level) covering the middle cingulate gyrus. Similarly, two clusters showed a significant leftward asymmetry, equally covering large parts of the hemisphere: the first cluster comprised 34,094 voxels (*p* ≤ 0.001, FWE-corrected on cluster-level) covering most of the hemisphere, while the second cluster comprised 671 voxels (*p* = 0.007, FWE-corrected on cluster-level) covering the inferior part of the cerebellar vermis.

### 3.2 Longitudinal changes in asymmetry over time (analysis II)

**Figure 2:**
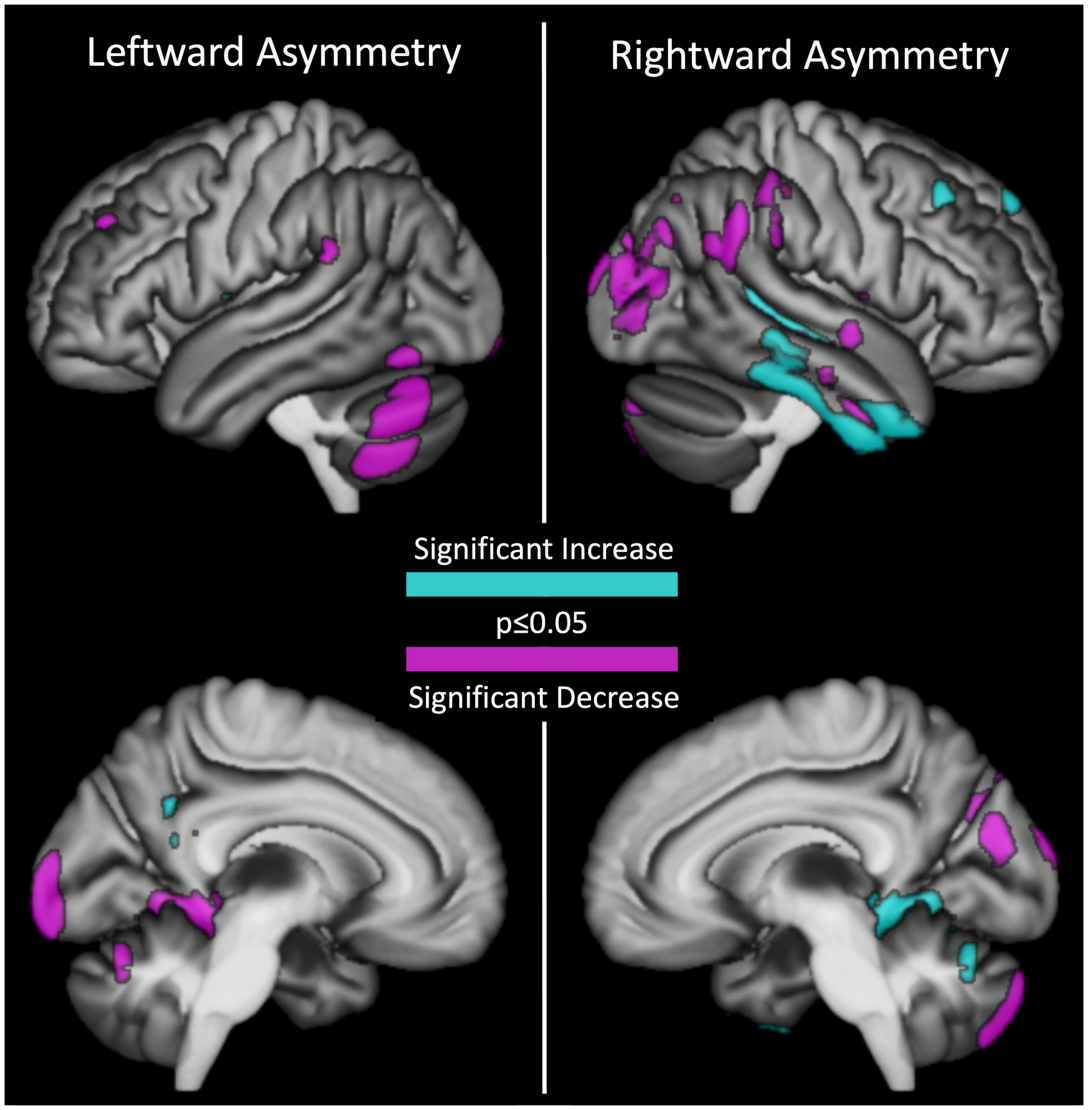
Regions with a significant change in gray matter asymmetry over time. Leftward asymmetries are shown on the left, rightward asymmetries are depicted on the right. Increases in asymmetry are depicted in cyan, decreases in asymmetry in pink. A switch in asymmetry (i.e., left-to-right or right-to-left) is shown in both hemispheres.

As shown in Figure 2, a confined set of regions – predominantly in the temporal and occipital lobe as well as the cerebellum – showed significant changes in asymmetry over 1–7 years (mean 2.39 years). Some of these regions showed a decrease in asymmetry (i.e., hemispheres become more similar over time), while others exhibited an increase in asymmetry (i.e., hemispheres become more different over time). Specifically, out of seven clusters with a leftward asymmetry at baseline, four decreased in asymmetry, one increased in asymmetry, and two switched from a leftward to a rightward asymmetry. Similarly, out of eight clusters with a rightward asymmetry at baseline, three decreased in asymmetry, four increased in asymmetry, and one switched from a rightward to a leftward asymmetry. Details for all clusters are given in Table 1. Additional cluster-specific information is provided in Supplementary Figure S1. Neither age nor sex had a significant effect on changes in asymmetry.

**Table 1:** Significant changes in asymmetry.

Cluster number	Size (voxels)	Significance (p_FWE-corrected_)	Nature of change	*x*	*y*	*z*	Brain region
Decrease in asymmetry							
1	1,780	<0.001	Leftward decrease	48	−59	−54	Lateral cerebellar hemisphere
2	752	0.006	Leftward decrease	9	−98	4	Occipital pole
3	569	0.002	Leftward decrease	44	−29	10	Heschl gyrus Planum temporale
4	328	0.039	Leftward decrease	35	42	34	Middle frontal gyrus
5	7,253	<0.001	Rightward decrease	25	−96	−5	Lateral occipital and parietal cortex
6	1,381	0.002	Rightward decrease	10	−84	−45	Posterior cerebellar hemisphere
7	1,233	<0.001	Rightward decrease	45	−1	−35	Anterior inferior and superior temporal sulcus
Increase in asymmetry							
8	603	0.016	Leftward increase	10	−52	32	Posterior cingulate gyrus
9	1,984	<0.001	Rightward increase	51	−5	−46	Anterior inferior temporal gyrus
10	933	<0.001	Rightward increase	44	−31	1	Superior temporal sulcus
11	338	0.001	Rightward increase	39	28	42	Middle frontal gyrus
12	275	0.007	Rightward increase	13	52	42	Superior frontal gyrus
Switch in asymmetry							
13	2,682	<0.001	Leftward to rightward	20	−37	−11	Hippocampus Inferior occipital cortex Superior cerebellar cortex
14	357	0.005	Leftward to rightward	29	16	6	Frontal pole
15	1,282	<0.001	Rightward to leftward	42	−23	24	Fronto-parietal operculum

## 4 Discussion

This study investigated whether and how gray matter asymmetry changes over time in a large sample of more than 2,000 participants. We observed significant changes of both leftward and rightward asymmetry, with increases, decreases, and even reversals of asymmetry. Importantly, these findings demonstrate that asymmetry changes are region-specific and follow distinct trajectories, providing novel insights beyond prior cross-sectional studies. Interestingly, asymmetry changes remained broadly stable, showing no acceleration or deceleration with increasing age, and did not differ between men and women. This generally linear pattern suggests that asymmetry changes may reflect differential but proportional declines across the two hemispheres rather than accelerated loss in one hemisphere alone. Together, these results indicate that changes in asymmetry are a typical feature of brain aging, following a largely stable trajectory with minimal sex differences.

### 4.1 Changes in asymmetry with increasing age

Reports on structural asymmetry within the framework of aging exist, but the underlying studies were cross-sectional in nature, correlating age and brain asymmetry with respect to various morphological measures. For example, one study (*n* = 70) examining gray matter in selected frontal and mesial temporal regions reported an absence of significant correlations between age and brain asymmetry (Smeets et al., 2010). Similarly, a study (*n* = 485) examining gray matter in Brodman Areas (BA) 44 and 45 reported an absence of significant correlations for BA 44, but a significant negative correlation between age and the rightward asymmetry of Brodman Area 45 (Kurth et al., 2020). Another study (*n* = 215) examining cortical thickness reported a significant positive correlation between age and the leftward asymmetry of the temporo-occipito-parietal cortex as well as between age and the rightward asymmetry of mesial parietal regions (Plessen et al., 2014). Yet another study (*n* = 15,847) examining subcortical brain volumes reported a significant positive correlation between age and the leftward asymmetry of the putamen (Guadalupe et al., 2017). When examining various cortical measures, another study (*n* = 17,141) reported a significant negative correlation between age and the rightward asymmetry of the superior temporal gyrus, with respect to cortical thickness, as well as a positive correlation between age and the leftward asymmetry of the superior temporal sulcus, with respect to cortical surface area (Kong et al., 2018). Moreover, when only including samples that spanned an age range of more than 20 years, the same study reported a significant positive correlation between age and the leftward asymmetry in overall cortical thickness, as well as a significant negative correlation between age and the leftward asymmetry in the surface area of the entorhinal cortex (Kong et al., 2018). The comparability between the outcomes of those studies and our current findings is somewhat limited, given the different analysis designs (e.g., longitudinal versus cross-sectional) or morphometric measures (e.g., voxel-wise gray matter versus regional surface area). Notwithstanding, the current findings – just as previous results – support the notion that age-related changes in asymmetry do not reflect a uniform shift towards a more (or less) asymmetric brain, but rather differ in their directionality across brain regions. Such region-specific patterns may reflect differential vulnerability of homologous left and right hemisphere regions to intrinsic aging processes and external influences.

### 4.2 No modulating impact of age and sex

Interestingly, there was no significant effect of age on asymmetry changes over time, indicating that changes are relatively stable without accelerating or decelerating with increasing age. While this might seem to contrast with reports of accelerating gray matter loss with increasing age (Coupe et al., 2017; Fjell et al., 2009; Fjell et al., 2013; Pfefferbaum et al., 2013; Ziegler et al., 2012), it is not. Note, our study examined changes in asymmetry of gray matter, not changes in gray matter per se. In other words, brain regions with accelerated gray matter loss, as reported in other studies, might not necessarily show an accelerated change in asymmetry as well (e.g., if homologous regions in both hemispheres decline at the same accelerated rate). In fact, the absence of accelerated changes in asymmetry supports previous reports suggesting that non-linear age effects are of little relevance (see Kong et al., 2022). This may also have functional relevance, with better preserved cognitive function during typical aging, compared to pathological aging. Indeed, there are reports of rapidly increasing asymmetries in early dementia (Cherbuin et al., 2010; Thompson et al., 2003), while the change in asymmetry remains stable over time in our study of relatively healthy participants.

Similarly to age, there was no significant effect of sex on asymmetry changes over time. In other words, male brains do not significantly become more (or less) asymmetric over time than female brains. This might seem surprising at first because biological sex is a major modulator of both, brain asymmetry (Guadalupe et al., 2017; Kong et al., 2018; Kurth et al., 2018; Luders and Toga, 2010; Ocklenburg and Gunturkun, 2024b) and brain aging (Austad, 2019; Dubal et al., 2025; Jahanshad and Thompson, 2017). However, while females and males might exhibit regional differences in asymmetry as well as regional differences in aging trajectories, such sex differences do not necessarily result in sex-specific changes in asymmetry over time.

### 4.3 Conclusion and outlook

The outcomes of our study support the notion of region-specific changes in asymmetry over time, which seem to be independent of age and sex. However, future studies are needed to confirm the current results. Moreover, the current study focused on voxel-wise gray matter volume, so expanding the range of morphometric measures would be desirable (e.g., cortical thickness, cortical surface area, cortical, and subcortical volumes), not only to provide a better comparability to previous studies but also to reveal if asymmetry changes are different or similar for different brain features. Optimally, such studies would include more than one follow-up as well as longer observation periods than only a few years. This would allow investigating actual trajectories of change over time and also relating any structural changes in asymmetry to corresponding functional processes in the framework of aging, including measures of cognitive reserve, brain health, as well as overall health (just to name a few). In the present work, we deliberately restricted our analyses to structural measures of asymmetry to establish a clear and foundational characterization of its longitudinal trajectories. While the UK Biobank indeed offers a wealth of phenotypic data, including cognitive, lifestyle, and health-related measures, incorporating these was beyond the scope of this initial investigation. However, the integration of such variables might be the next critical step that would possibly not only clarify the functional significance of asymmetry changes but also link them more directly to individual differences in cognition, lifestyle, and health. Finally, future studies might consider contrasting asymmetry changes in healthy populations with those in clinical populations (e.g., individuals with age-related neuropathologies) to address whether asymmetry changes are accelerated over time in patients or whether disease severity, symptom progression, or treatment response is associated with (accelerated) changes in asymmetry. Last but not least, future studies may also want to include measures of structural and functional connectivity as well as cognitive measures building on (and potentially expanding) existing models of aging that propose a change of structural asymmetry or functional lateralization due to an adaptive reorganization to counteract effects of age-related atrophy. For example, the HAROLD theory proposes that homologous areas in the opposite hemisphere are progressively recruited with increasing age (Cabeza, 2002; Dolcos et al., 2002), while further refinements of that theory argue that recruitment of additional brain regions only occur at higher cognitive load with recruitment of a potentially larger network of regions not necessarily restricted to homologous areas in the contralateral hemispheres (Berlingeri et al., 2013; Park and Reuter-Lorenz, 2009; Reuter-Lorenz and Park, 2008).

## Data Availability

All data were obtained from the UK Biobank.

## Footnotes

^1^Clusters that changed in asymmetry from left to right (or from right to left) are projected onto both hemispheres.
