# Supplementary material for: Changes in voxel-wise gray matter asymmetry over time
Source: Front Neurosci. 2025 Dec 1;19:1671341. doi: 10.3389/fnins.2025.1671341 (PMC12702909; doi:10.3389/fnins.2025.1671341)
Supplement: Supplementary file 1 [file Data_Sheet_1.PDF]

Supplementary Figure 1

Cluster-specific gray matter asymmetry. Left panel: Gray matter asymmetry at baseline and follow-up. The bar depicts the mean, the whiskers depicts the standard deviation. Right panel: Change in gray matter asymmetry over time. The boxes depict the median and the inter-quartile range, the whiskers depict the 1.5 interquartile range. The dots are the individual changes.
Note, cluster numbers corresponds to Table 1. Decreases in asymmetry are shown in pink, increases in cyan, in correspondence with Figure 1.

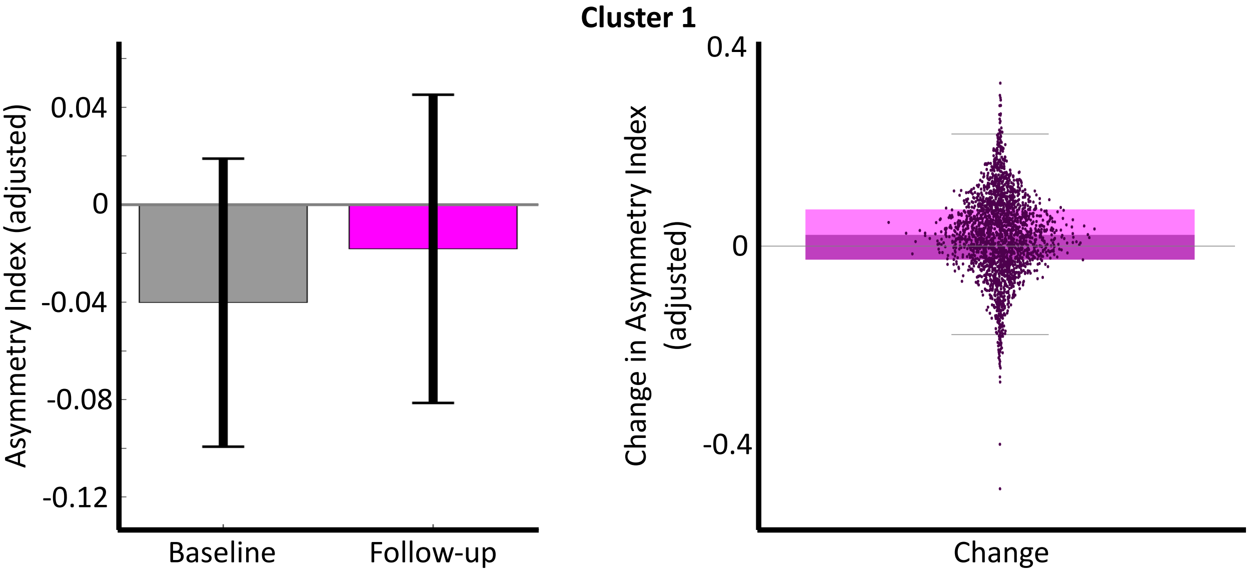

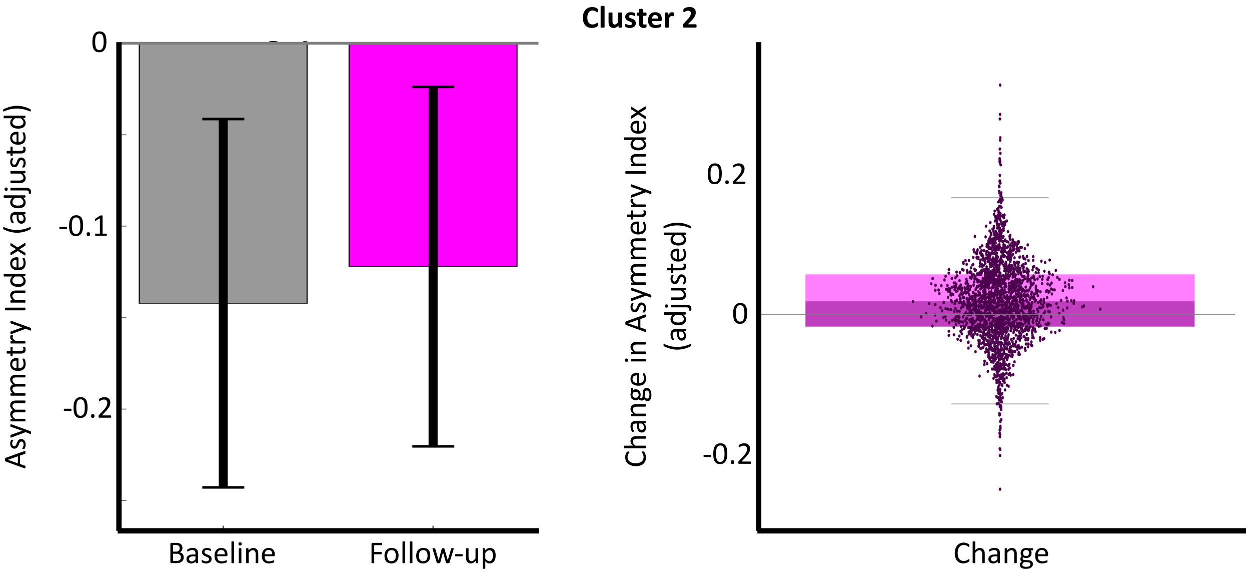

Cluster 3

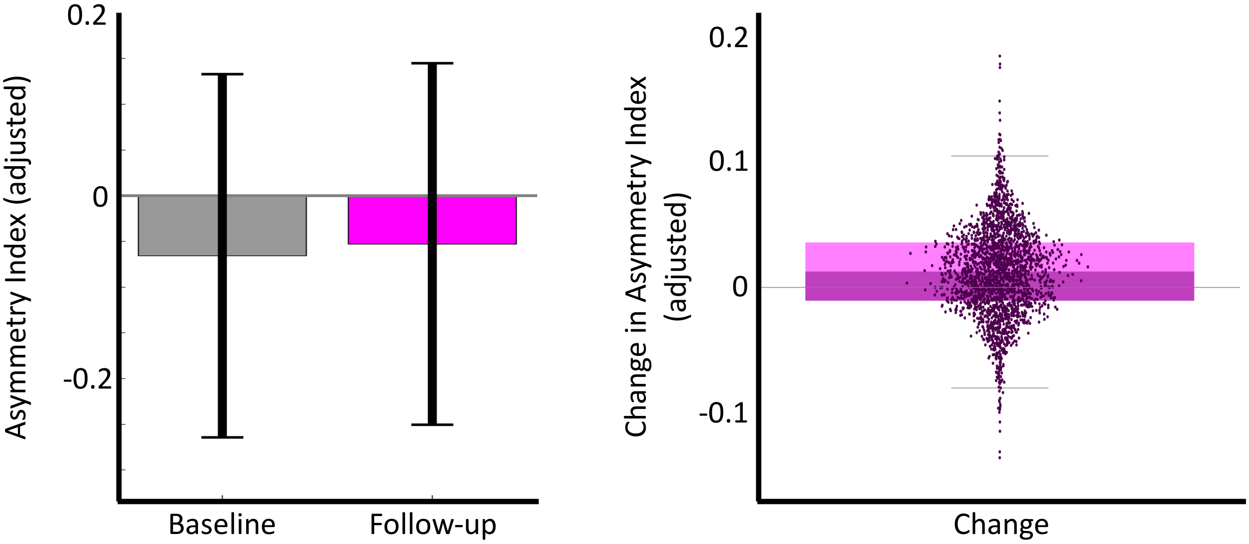

Cluster 4

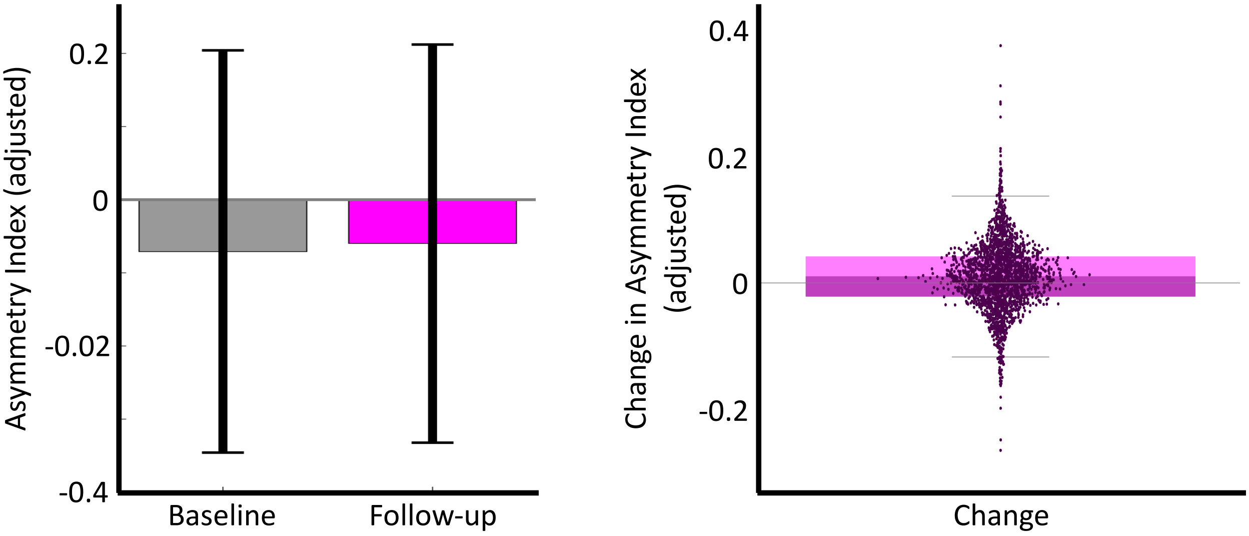

Cluster 5

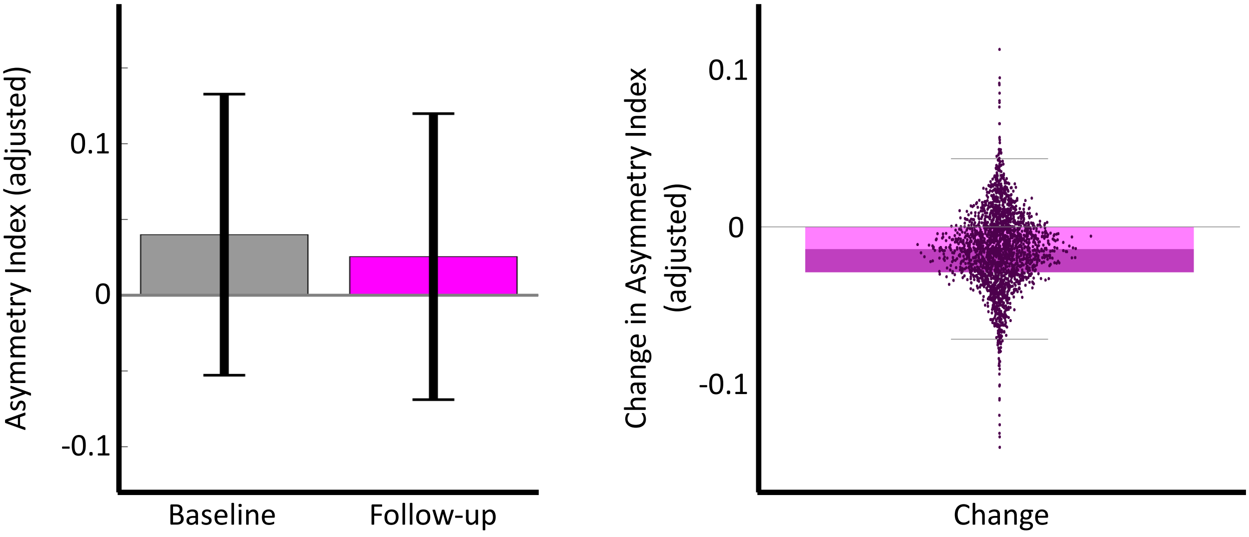

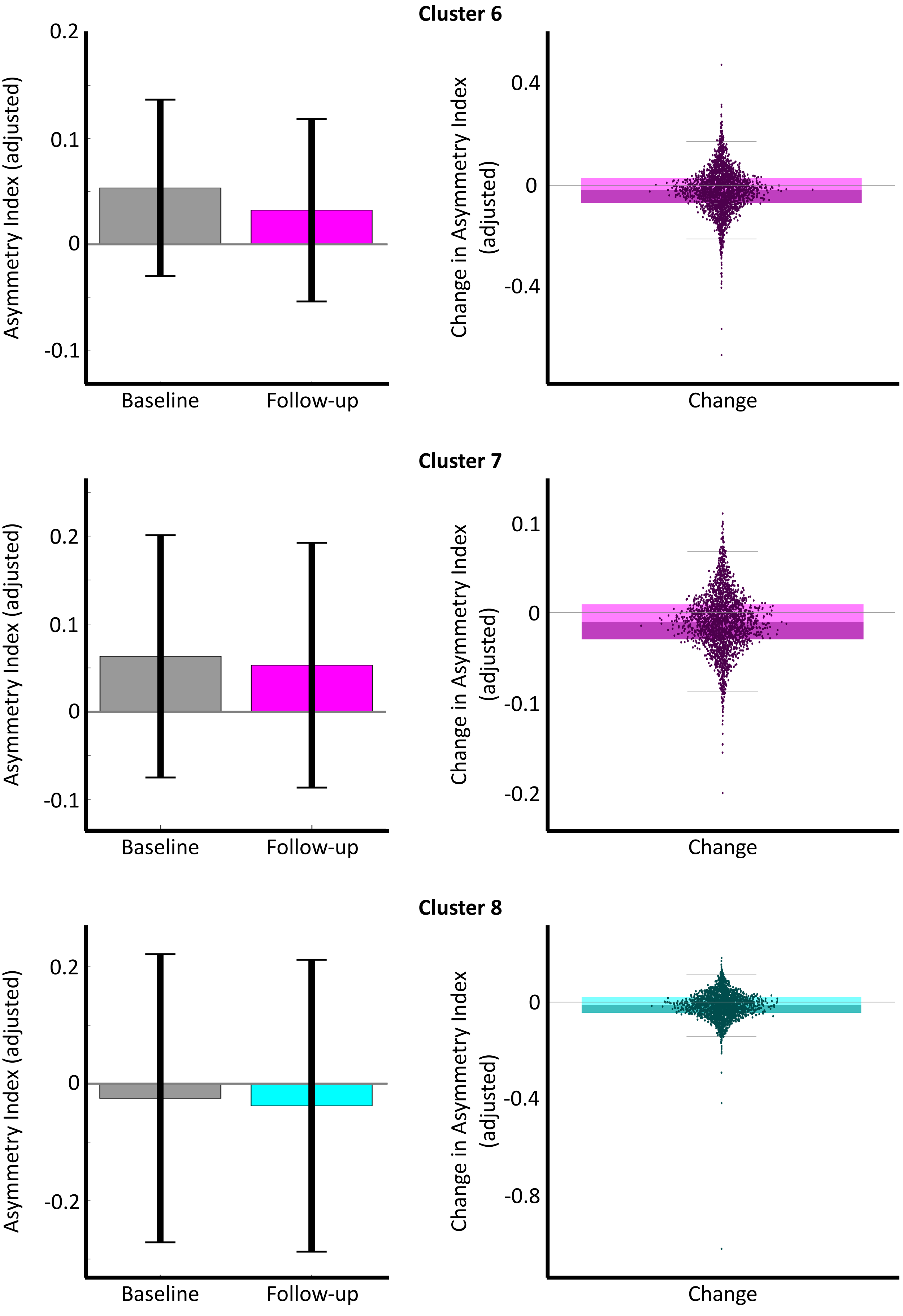

Cluster 6
Asymmetry Index (adjusted)
0.2
0.1
0
-0.1
Baseline
Follow-up
Change in Asymmetry Index (adjusted)
0.4
0
-0.4
Change
Cluster 7
Asymmetry Index (adjusted)
0.2
0.1
0
-0.1
Baseline
Follow-up
Change in Asymmetry Index (adjusted)
0.1
0
-0.1
-0.2
Change
Cluster 8
Asymmetry Index (adjusted)
0.2
0
-0.2
Baseline
Follow-up
Change in Asymmetry Index (adjusted)
0
-0.4
-0.8
Change

Cluster 9

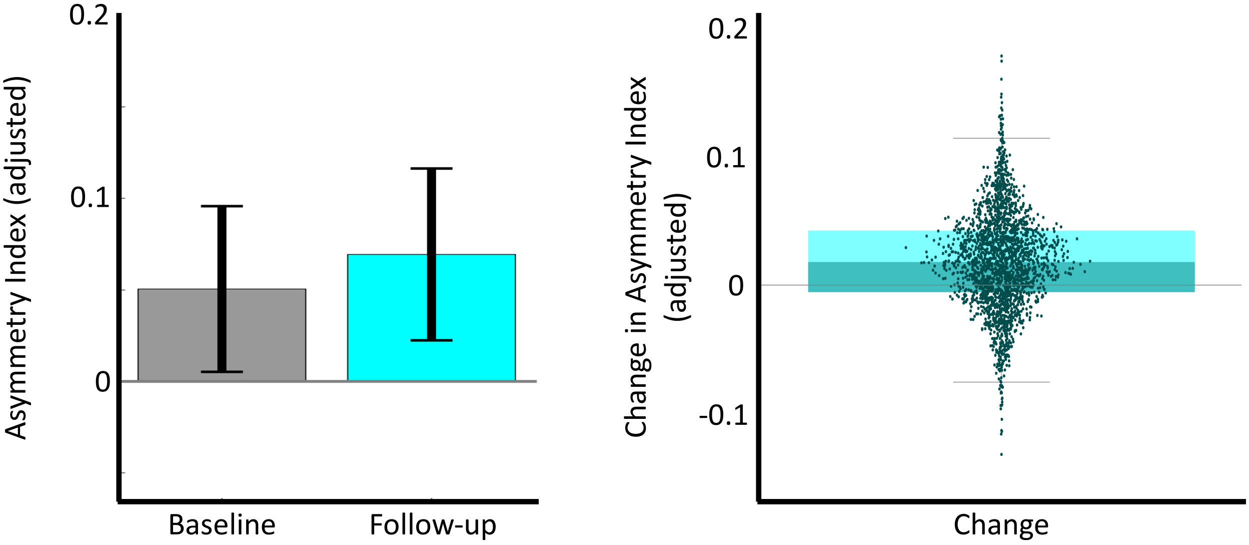

Cluster 10

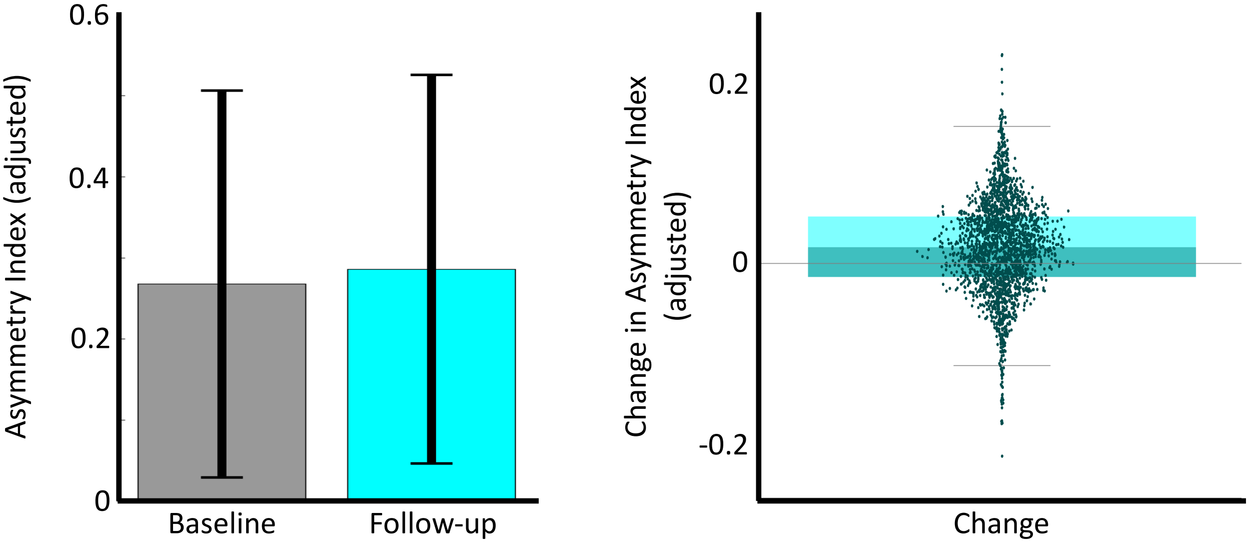

Cluster 11

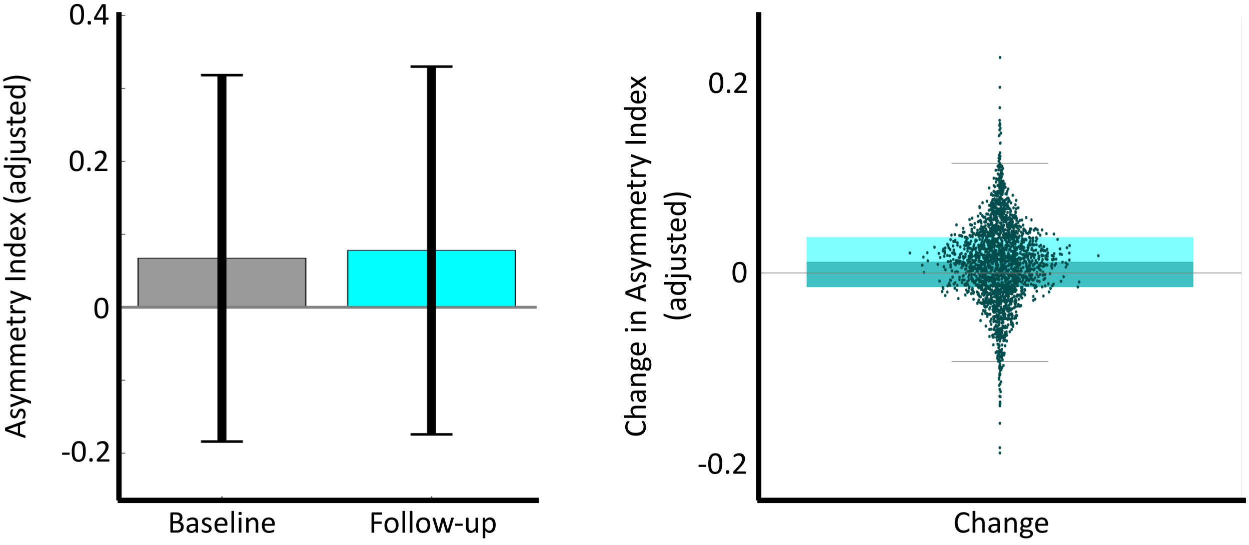

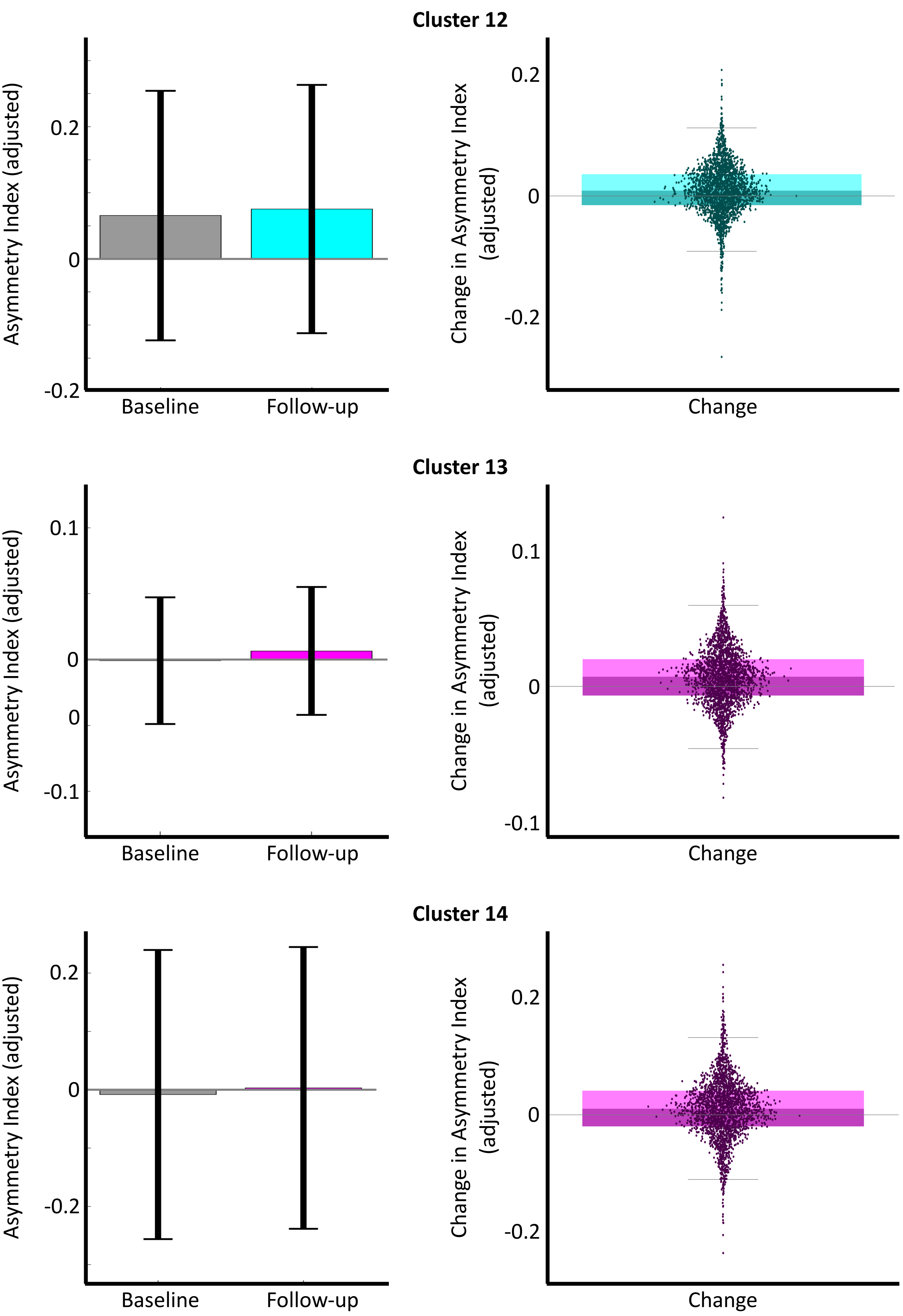
Cluster 12
Asymmetry Index (adjusted)
0.2
0
-0.2
Baseline
Follow-up
Change in Asymmetry Index (adjusted)
0.2
0
-0.2
Change
Cluster 13
Asymmetry Index (adjusted)
0.1
0
0
-0.1
Baseline
Follow-up
Change in Asymmetry Index (adjusted)
0.1
0
-0.1
Change
Cluster 14
Asymmetry Index (adjusted)
0.2
0
-0.2
Baseline
Follow-up
Change in Asymmetry Index (adjusted)
0.2
0
-0.2
Change

Cluster 15

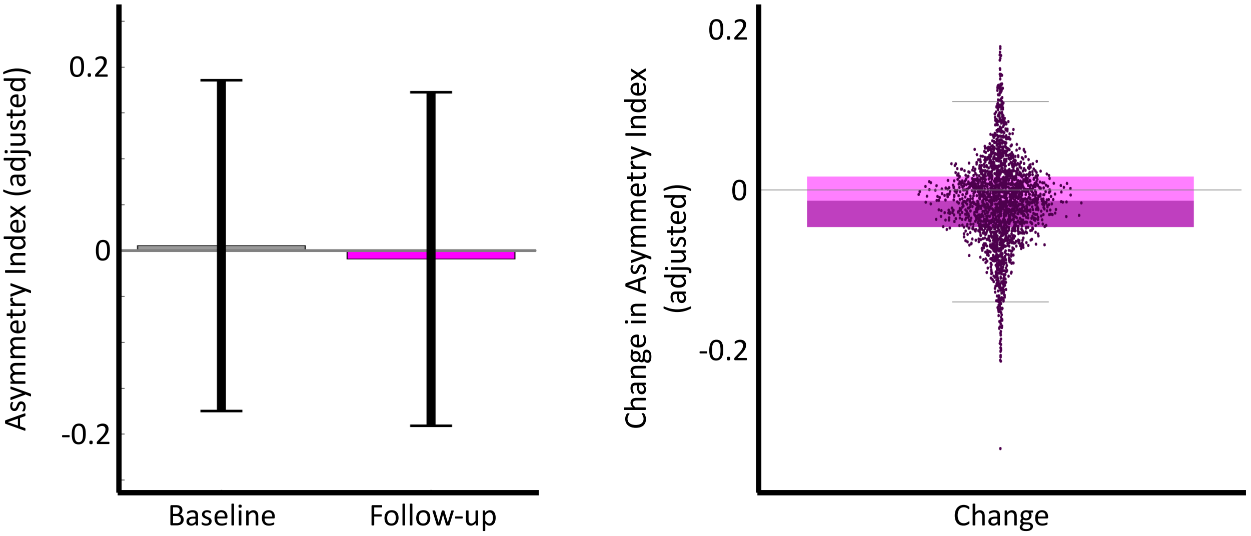

Asymmetry Index (adjusted)
0.2
0
-0.2
Baseline
Follow-up
Change in Asymmetry Index (adjusted)
0.2
0
-0.2
Change
